# Supplementary material for: NatB Catalytic Subunit Depletion Disrupts DNA Replication Initiation Leading to Senescence in MEFs
Source: Int J Mol Sci. 2023 May 13;24(10):8724. doi: 10.3390/ijms24108724 (PMC10218504; doi:10.3390/ijms24108724)
Supplement: Supplementary file 1 [file ijms-24-08724-s001.zip › ijms-2380599-supplementary.pdf]

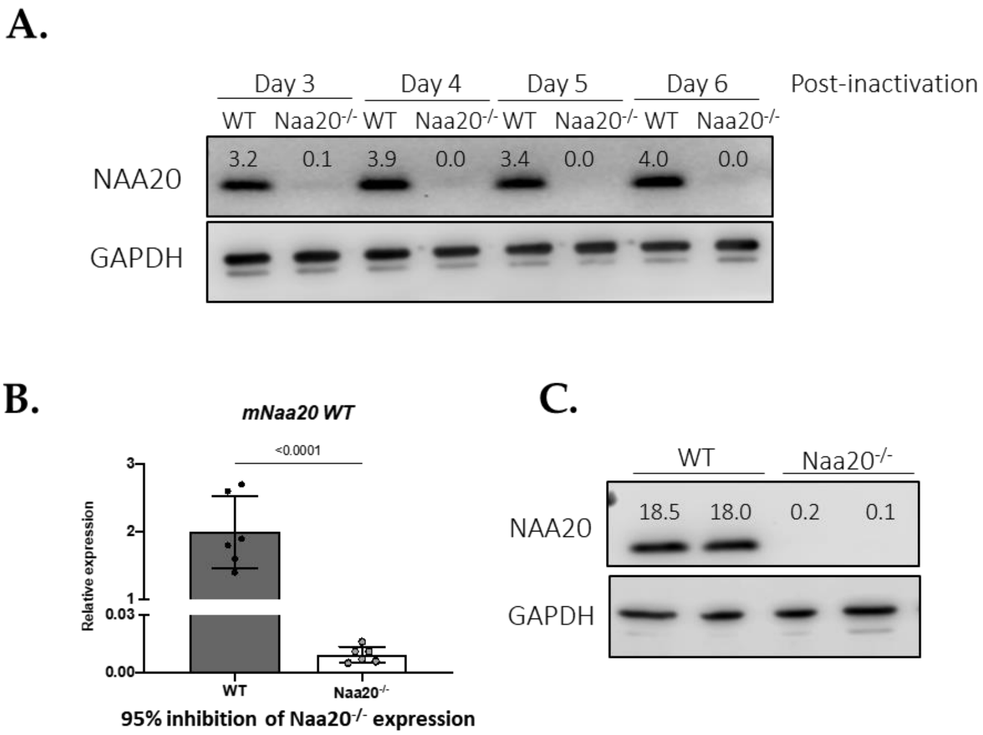

Figure S1. Analysis of Naa20 protein levels in Naa20 MEFs. (**A**) Western blot analysis of cell extracts of Naa20 MEFs infected with AdEmpty (WT) and AdCre (Naa20$^{-/-}$) 3-, 4-, 5- and 6-days post-infection. (**B**) Quantitative real time PCR for Naa20 wild type allele mRNA expression analysis relative to *GAPDH* and (**C**) western blot analysis of Naa20 in Naa20WT and Naa20$^{-/-}$ MEFs 6 days post-inactivation. The intensity values of each band are indicated.

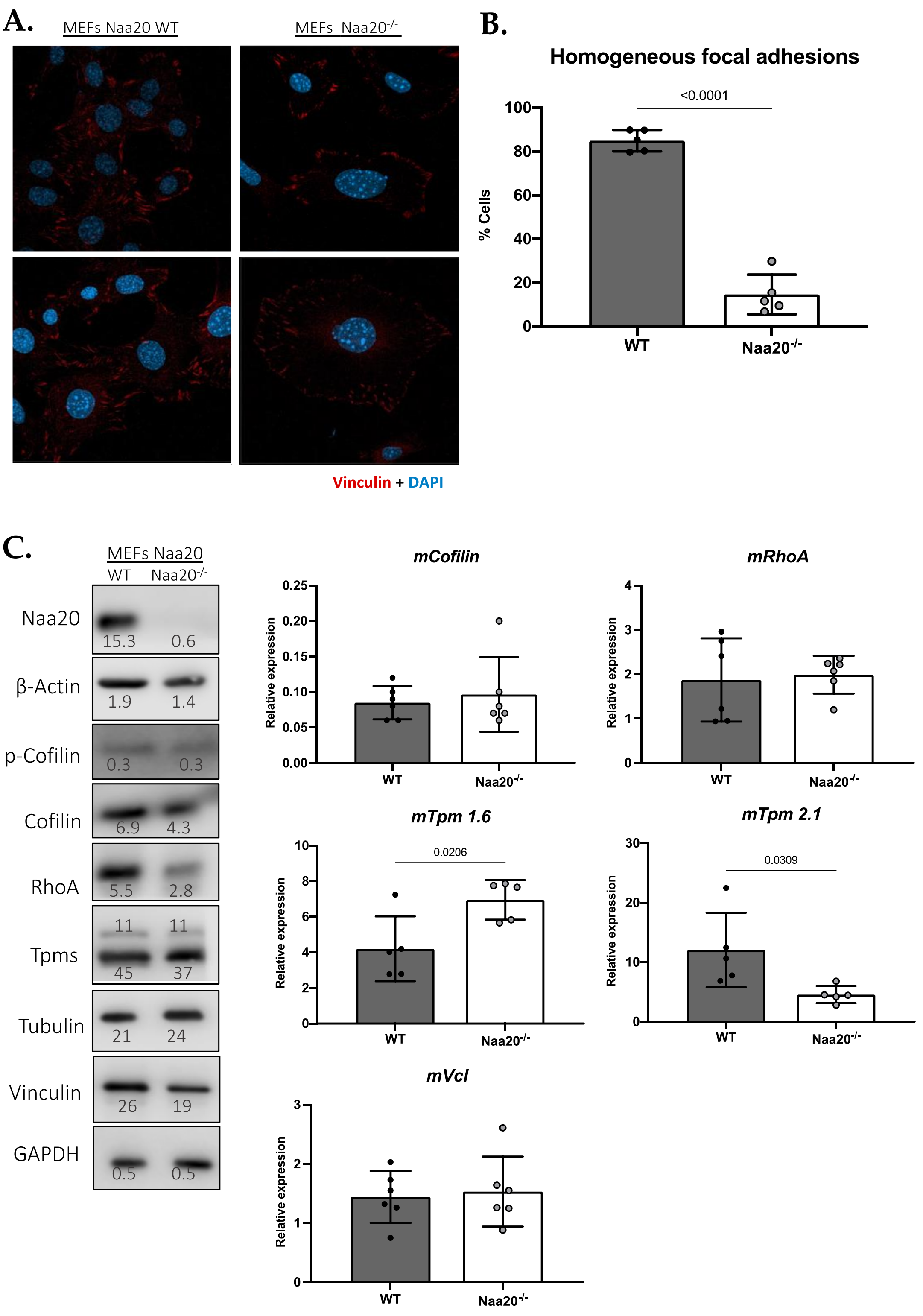

Figure S2. Actin cytoskeleton structure analysis in Naa20 MEFs. (**A**) Representative immunofluorescences of WT and Naa20$^{-/-}$ MEFs of focal adhesions (vinculin, red) and nuclei (DAPI, blue). The intensity values of each band are indicated. (**B**) Quantification of the number of cells with focal adhesions homogeneously distributed in the center and periphery of the cells. Cells with focal adhesions just at the periphery are considered as negative. Means of 5 independent experiments are represented in the graph. (**C**) Western blot analysis of Naa20 MEFs protein extracts 6 days post-inactivation. Quantitative real time PCR expression analysis of actin

cytoskeleton components relative to *GAPDH* in Naa20WT and Naa20$^{-/-}$ MEFs 6 days post-inactivation. In all the graphs the p values were calculated by unpaired T-test; mean ± SD are represented.

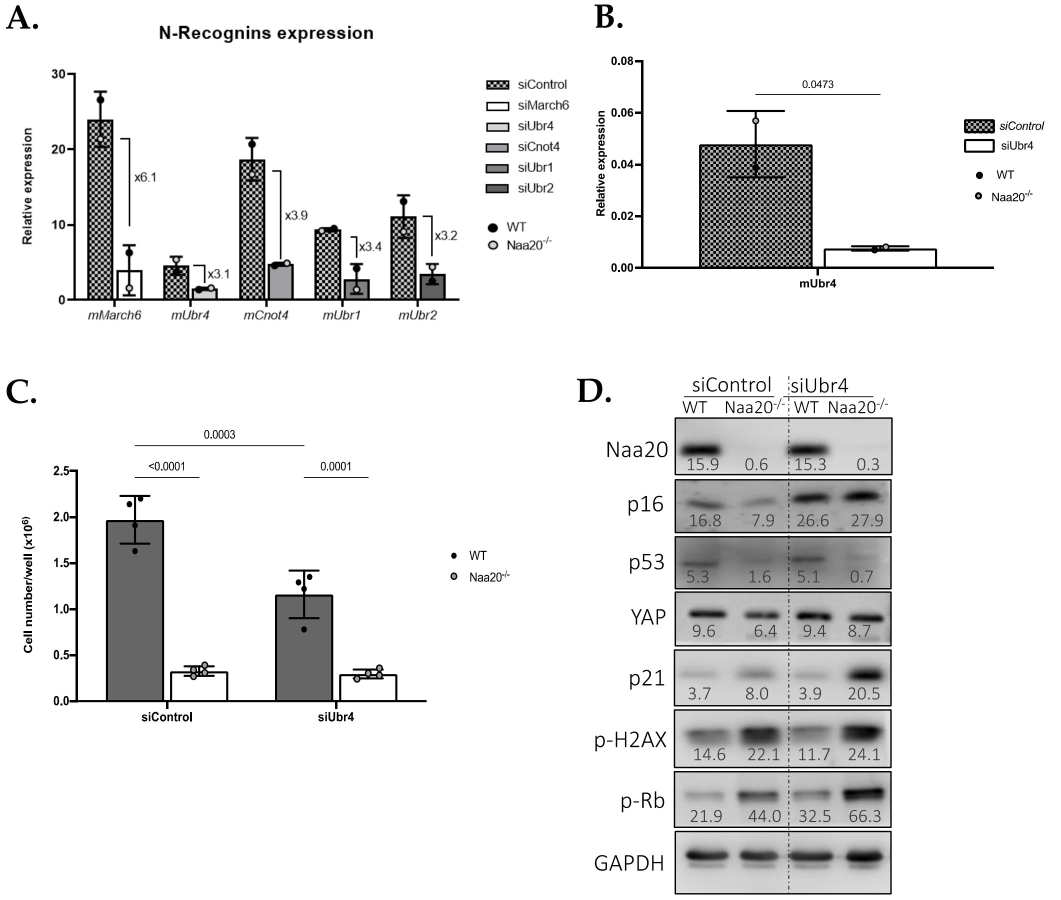

Figure S3. N-degron pathway analysis in Naa20 MEFs. (**A**) Gene expression analysis of N-recognins after downregulation with specific siRNAs. qRT-PCR of *mMarch6*, *mUbr4, mCnot4, mUbr1, and mUbr2 genes*. Fold change was calculated with ration siN-recgonin/siControl; mean ± SD are represented. (**B**) Gene expression analysis of N-recognin *mUbr4* after downregulation with a specific siRNA. Mean ± SD are represented. (**C**) Proliferation analysis of WT and Naa20$^{-/-}$ MEFs after Ubr4 silencing 6 days post Naa20 inactivation. A specific siRNA (siControl) was used as control. (**D**) Western-blot analysis of G1 to S transition components in WT and Naa20$^{-/-}$ MEFs after siControl (siRNA Silencer™ Select Negative Control No. 1 (Thermo Scientific, 4390843)) or siUbr4 transfection 6 days post Naa20 inactivation. p values were calculated by two-way ANOVA analysis; mean ± SD are represented. The intensity values of each band are indicated.

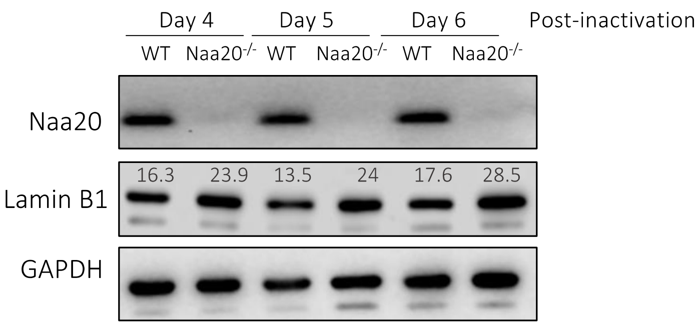

Figure S4. Analysis of Lamin B1 protein levels in Naa20 MEFs. Western blot analysis of cell extracts of Naa20 MEFs infected with AdEmpty (WT) and AdCre (Naa20$^{-/-}$) 4-, 5- and 6-days post-infection. The intensity values of each band are indicated.
